# A simple practical patient-reported clinic satisfaction measure for young adults

**DOI:** 10.1038/sj.bjc.6605923

**Published:** 2010-11-09

**Authors:** R Phillips, K Absolom, D Stark, A Glaser

**Affiliations:** 1 Department of Paediatric Oncology and Haematology, Leeds Teaching Hospitals Trust, Beckett Street, Leeds LS9 7TF, UK; 2 Psychosocial Oncology and Clinical Practice Research Group, St James's Institute of Oncology, Bexley Wing, Beckett Street, Leeds LS9 7TF, UK

**Keywords:** patient reported outcome measure, validation, patient satisfaction questionnaire

## Abstract

**Background:**

The development of practical patient-reported outcome measures (PROM) to assess the user view of health programmes is increasingly important. Valid, shorter instruments are more likely to be used and completed than extensive questionnaires.

**Methods:**

Consecutive adult outpatient attendees who were long-term survivors of childhood cancer completed the 16-item Patient Satisfaction with Communication Questionnaire (PSCQ). These data were used to develop a three-item questionnaire. The brief PROM was validated against data from a second, independent survey conducted in a similar fashion.

**Results:**

In all, 93 individuals contributed PSCQ data, a response rate of 63%. The brief PROM was highly correlated with the original PSCQ in derivation (*ρ*=0.87, *P*<0.001) and validation (*ρ*=0.82, *P*<0.001) data sets. Using a cutoff of scores <9 to indicate dissatisfaction showed fair discrimination in derivation (sensitivity 85%, specificity 80%) and validation data sets (sensitivity 75%, specificity 78%).

**Conclusion:**

It is possible to quickly and efficiently assess satisfaction with follow-up clinics with three questions. This brief PROM could prove useful in monitoring services quality by allowing clinic users to provide timely feedback on their care.

NHS service providers are mandated to assess and respond to the user view with respect to the health programmes they deliver (Department of Health, 2009). Good quality patient-reported outcome measures (PROM) are urgently needed (Department of Health, 2009; Department of Health, 2010); these should improve the quality of services and will have direct financial implications (Department of Health, 2009).

Adult survivors of childhood cancer are likely to experience one or more significant problems in later life (Oeffinger *et al*, 2006). As such they are representative of populations living with chronic illness, with the potential for long-term and ongoing health and social care needs. Provision of long-term follow-up programmes is mandated to define and address the emerging health needs of this group of survivors (National Institute of Clinical Excellence, 2005).

This study describes the development of a PROM to assess satisfaction with long-term follow-up consultations for adult survivors of childhood cancer. The measure is derived from a previously validated questionnaire (Shilling *et al*, 2003) with the aim of shortening the original tool for practical purposes and ease of administration.

## Methods

### Setting

The study was conducted over two six-month periods between October 2007 to March 2008 and October 2008 to March 2009 in a large regional childhood cancer centre in the North of England, with patients receiving care in either a Paediatric Outpatient facility or a new Adult Cancer Centre. Satisfaction data were collected as part of a larger questionnaire-based assessment of follow-up services, which focussed on the validation and development of proposed models of ‘successful’ long-term follow-up care. The study was granted Ethical approval by the Leeds East PCT Ethics Review committee (REC application 07/H1306/116).

### Eligibility criteria and procedure

Consecutive patients were invited to participate if they were 18 years of age or older, had been diagnosed with a cancer or received a bone marrow transplant for a non-malignant condition before their 18th birthday and were at least 5 years from completion of therapy. They were required to be fluent in English and able to complete the questionnaire.

Clinic satisfaction questionnaires were given at the end of routine appointments, and returned in stamped addressed envelopes. A single reminder questionnaire was posted to non-responders after 4–6 weeks.

### Measure

Satisfaction was assessed using the Patient Satisfaction with Communication Questionnaire (PSCQ). This measure was developed by Fallowfield and colleagues (Shilling *et al*, 2003) and has been previously used with young adult cancer survivors (Absolom *et al*, 2006). The 16-item questionnaire measures consultation satisfaction in relation to three factors: (1) rapport (*n*=6, *the staff; answered all questions, seemed to know what they are doing, handled the consultation well, did their best to keep me from worrying, told me what I wanted to know; seemed sympathetic*); (2) manner (*n*=6, *the staff; would have been irritated if I’d asked too many questions, could have been more respectful, were too business like and impersonal, lacked experience with my medical problems, made me feel awkward, more attention could have been made to my privacy*); (3) understanding (*n*=4, *the staff used medical terms I didn’t understand, told me all there was to know, I feel unclear about some of the things the doctor told me, I am satisfied with the medical care I received*). Each statement is scored on a five-point categorical scale, from ‘strongly agree’ through ‘neither agree nor disagree’ to ‘strongly disagree’ 1–5 or 5–1 depending on positive or negative phrasing. The total score ranges from 16 to 80 with higher scores indicating greater satisfaction.

### Statistical analysis

In an attempt to shorten the PSCQ, a factor analysis using the principle factor method (unrotated) was undertaken. Factors with the highest loading were then used to create a brief questionnaire, and the ability of this to predict the ‘full’ satisfaction score was tested using bootstrapped linear regression and calculation of Pearson's correlation coefficient. In recognition of the overoptimistic nature of exploratory analyses, all coefficients were ‘shrunk’ to unity (Dawes, 1979; Harrell *et al*, 1996). To further improve the practicality of this brief measure, its ability to identify those patients in the bottom decile of overall satisfaction scores was explored in a dichotomous cutoff set, which maximised the sensitivity and specificity. The comparisons of demographics between groups were undertaken by *χ*^2^-tests for categorical variables and Kruskal–Wallis or *t*-tests for continuous variables as appropriate. All statistical analyses were undertaken with Stata10 (StataCorp, 2007).

The brief PROM was then assessed in a second independent, previously published, data set. This study enroled 198 of long-term survivors of childhood cancers from two centres in Yorkshire with the aim to describe and identify key variables, which explained patient satisfaction. It used the same outcome questionnaire and had very similar inclusion criteria (Absolom *et al*, 2006).

## Results

### Patients

In all, 143 of 173 eligible patients entered the study, 69 from the Paediatric and 74 from the Adult Cancer Centre (Table 1). Completed PSCQs were received from 93 individuals (response rate 65%). Fewer males than females returned questionnaires (52 *vs* 76%, *P*=0.02), but there were no significant differences by clinic location, cancer type or age.

### Satisfaction score

The overall measurement of satisfaction was significantly right-skewed, as expected from this type of measure. The median satisfaction score was 67 (range 44–78). This did not vary by duration of survival (Pearson's *ρ*=0.06, *P*=0.57) location, cancer type or age, but women reported a higher mean score (Table 2).

### Short satisfaction score

Factor analysis of the total satisfaction score revealed a single principal factor accounting for 67% of the variance. Three items loaded onto this factor with weights >0.72. A fourth item loaded with weight 0.68, and others were <0.65. The highest loading items were examined and a brief questionnaire was constructed with unitary weighting of the items, giving potential scores from 3 to 15. The three-item questionnaire and overall satisfaction score were highly correlated (Pearson's *ρ*=0.87, *P*<0.001, 95% CI 0.80–0.92). The median score of the brief questionnaire was 11 (range 4–12).

**Figure 1:**
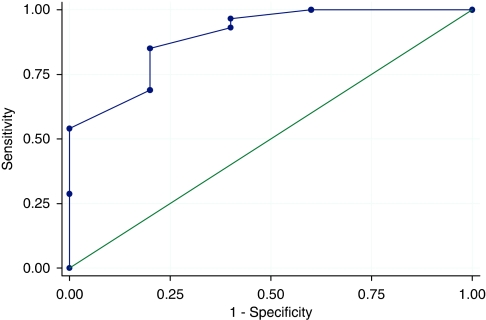
Receiver operator curve of the brief questionnaire to identify the least satisfied decile of patients. Area under the ROC curve=0.8707.

The ability of the score to identify the lowest decile of satisfaction scores was examined using receiver operator curve analysis (Figure 1). A cutoff at ⩾9 was most efficient, with a sensitivity of 85% and specificity of 80% (likelihood ratio (9 or more)=4.25, (8 or less)=0.18), meaning only 2% of patients with scores of nine or more were in the ‘dissatisfied’ decile.

The brief questionnaire consists of three questions, all commencing with the same stem and answered as a five-point categorical scale, from ‘strongly agree’ through ‘neither agree nor disagree’ to ‘strongly disagree’. The questions are:

The staff I saw at the clinic

- …used medical terms that I didn’t understand (scored 1–5)
- …did their best to keep me from worrying (scored 5–1)
- …would have been irritated if I’d asked too many questions (scored 1–5)

### Testing the questionnaire

The brief questionnaire was then tested by comparing the scores with the total satisfaction from the independent data set. As reported previously, this had a median satisfaction score of 64 (range 43–80), a mean age of 23.7 years and 52% female participants (Absolom *et al*, 2006).

**Figure 2:**
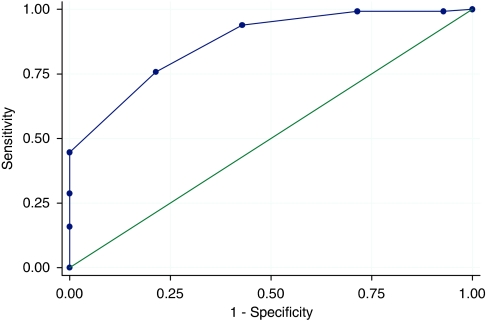
Receiver operator curve of the brief questionnaire to identify the least satisfied decile of patients in the validation set. Area under the ROC curve=0.8707.

In this ‘validation’ data set, the brief questionnaire again provided good correlation with the overall score (Pearson's *ρ*=0.82, *P*<0.001, 95% CI 0.76–0.87: comparing validation and derivation sets, *P*=0.16). In this data set, the median score of the brief questionnaire was 9 (range 5–12). There was no systematic difference in correlation between the two centres (centre 1 *ρ*=0.81 (95% CI 0.74–0.89), centre 2 *ρ*=0.83 (95% CI 0.71–0.88), *P*=0.67). The ‘diagnostic’ ability of a cutoff of ⩾9 to identify the lowest decile was also comparable (sensitivity 75%, specificity 78%, scores of 9 or more, predictive value of 3% for ‘dissatisfaction’ Figure 2).

## Conclusion

Adult survivors of childhood cancer who attend a long-term follow-up programme are generally satisfied with their experience of outpatient clinics. In this study, women showed a greater satisfaction with care, the opposite of the previous study (Absolom *et al*, 2006), but this difference was small and both differences may well reflect the chance effects of random sampling. No clear difference between cancer types, duration of survival and location of care was found.

A brief questionnaire was derived from the original PSCQ data consisting of only three questions. This provided a highly accurate reflection of the overall satisfaction score and retained its predictive ability in a separate data set taken in both geographically and temporally diverse settings. The three elements of this short questionnaire originate from the three factors described in the original tool; (1) rapport ‘did their best to keep me from worrying’, (2) manner ‘would have been irritated if I’d asked too many questions’ and (3) understanding ‘used medical terms that I didn’t understand’. Overall these questions appear to demonstrate face validity in reflecting the key components of a satisfactory clinical encounter from communication perspective.

The derivation and use of very short clinically useful diagnostic measures has an extensive history in cancer (Mitchell, 2007) and more broadly, for example, in dementia (Holsinger *et al*, 2007) and alcohol misuse (Kriston *et al*, 2008). These measures, although losing some of the accuracy of the original tool, frequently retain the power to enable key clinical decisions to be made. Analogous work in the development of methodologies for clinical trials has demonstrated that shorter questionnaires are more likely to be returned, and completed in full, than longer versions (Edwards *et al*, 2007). Although some detailed information is lost using the short questionnaire, we believe this minimal reduction in data is more than balanced by the potential improvement in return rates, thus giving an overall more comprehensive and complete evaluation of the service under scrutiny.

This study provides support for this very short questionnaire, but has limitations: (1) the items were completed within a longer questionnaire (four pages of A4 paper in total, and included 13 other questions covering further elements of the outpatient experience). Administered independently, they may produce different effects. The estimate of the predictive ability is based within this setting, and may reflect overoptimism. To reduce this, the use of unitary weighting and bootstrap estimations of confidence intervals are presented. A further study using these three questions should be undertaken. (2) The study addressed the satisfaction of people who chose to attend clinic, and take part in the study they were offered. This particular population may not be reflective of the wider community, noting that there was a 63% return rate from the participants and those who did not return the PSCQ may systematically differ from those who did. (3) This questionnaire follows a traditional face-to-face clinical review, and may not be applicable in alternative immediate settings, such as telephone or telemedicine clinics, and will almost certainly be ineffective in staggered review settings, such as postal or email/web-based reviews. (4) The study demanded fluency in English language, therefore excluding some participants at the clinic who, it may be postulated, have the greatest risk of communication problems.

It should be acknowledged that patients successfully treated for a life threatening illness may be very loyal to their care teams and reluctant to criticise staff as a result. Therefore, when implementing the routine completion of satisfaction measures, encouraging honest feedback by asking patients to complete measures outside the clinic environment and by highlighting the confidentiality of their responses may be beneficial. In addition to this point, the measurement of consultation satisfaction addresses one aspect of follow-up care and additional assessments of how late effects are being detected and managed would be necessary to determine the overall success of a service.

It is possible to quickly and efficiently assess satisfaction with outpatient follow-up clinics using of three questions. This PROM could prove useful in monitoring and improving services while allowing clinic users to provide timely and valuable feedback on their care. The simplicity of this questionnaire is such that it could be completed as the patients left clinic, on paper, a touch screen computer or later by email or web-based form. There is an urgent need to confirm its usefulness, refine the approach if necessary, and embed quick, robust, user-friendly patient-reported clinic satisfaction assessments in every day practice.

## Figures and Tables

**Table 1:** Participant demographics

**Variable**	**Paediatric outpatient location (*N*=69)**	**Adult outpatient location (*N*=74)**	***P*-value**
Mean ages, years (s.d.)	24.9 years (4.5)	26.8 years (7.3)	0.29
Gender (female:male)	35:34	39:35	0.81
Mean survival, months (s.d.)	185 months (64.3)	211 months (84.8)	0.10
*Cancer type*			0.190
Leukaemia	22	19	
Lymphoma	13	18	
Brain tumour	13	7	
Other solid tumour	21	30	

**Table 2:** Mean satisfaction score by participant characteristics

**Paediatric clinic**	**Adult**	***P*-value**
64.45	66.65	0.195
		
**Female patient**	**Male**	
67.32	63.16	0.018
**Cancer type**		0.995
Leukaemia	65.8	
Lymphoma	65.7	
Brain tumour	65.1	
Other solid tumour	65.9	
